# Neuroanatomical Correlates of Binge-Eating Behavior: At the Roots of Unstoppable Eating

**DOI:** 10.3390/brainsci11091162

**Published:** 2021-08-31

**Authors:** Rossella Oliva, Sanja Budisavljević, Umberto Castiello, Chiara Begliomini

**Affiliations:** 1 Centro Terapia e Ricerca sui Disturbi Alimentari (Center for Eating Disorders Therapy and Research—CenTeR Disturbi Alimentari), 30172 Venice, Italy; rossellaoliva.psi@gmail.com; 2 School of Medicine, University of St Andrews, St Andrews KY16 9BA, UK; sanja.budisavljevic@gmail.com; 3 Department of General Psychology, University of Padova, 35131 Padova, Italy; umberto.castiello@unipd.it

**Keywords:** voxel-based morphometry, dorsolateral prefrontal cortex, impulsivity, binge-eating

## Abstract

Binge-eating refers to episodes of uncontrolled eating accompanied by a perceived loss of control, which can be common in the general population. Given the profound negative consequences of persistent binge-eating such as weight and eating disorders, it is vital to determine what makes someone more vulnerable than others to engage in such a conduct. A total of 42 normal-weight individuals (21 with binge-eating episodes and 21 without binge-eating episodes) underwent a structural magnetic resonance imaging measurement and Voxel-based morphometry (VBM) was used to assess between-group differences in terms of gray matter volume (GMV), together with self-report impulsivity and binge-eating measures. The results showed binge-eating individuals as characterized by higher trait impulsivity and greater regional GMV in the left middle frontal gyrus: however, the GMV in this region appeared to be positively correlated only with measures of binge-eating but not with trait impulsivity measures. These findings provide novel insights on the neurobiological roots of BE in normal-weight individuals and highlight how this behavior can be associated with brain morphometric changes within prefrontal regions also in a non-clinical population. Overall, this study provides a further characterization of the neural correlates of binge-eating and novel insights into the treatment of its more severe pathological forms.

## 1. Introduction

With the worldwide rise of overeating and overweight in the past few decades, researchers have put considerable attention on trying to understand the possible predisposing factors that may contribute to the development of obesity and binge-eating disorder (BED) [1]. The term binge-eating refers to a behavior characterized by episodes of uncontrolled eating of significant amounts of food in limited periods of time (American Psychiatric Association, APA) [2]. Within the general and non-clinical population, this behavior may become more frequent and compulsive over time, leading to the development of BED, weight gain, and obesity [3]. Hence, it becomes critical to understand what makes some individuals more prone than others to engage in such eating conduct.

Preliminary insights into binge-eating derive from a recent line of research according to which there would be a shared behavioral and neural substrate between overeating and substance compulsive use [4,5]; in both cases, a failure of inhibitory mechanisms and high impulsivity would play key roles in the tendency to engage in such behaviors despite the negative consequences [4,6]. Support to this argument derives from neuroimaging investigations that underscored the role of prefrontal cortex (PFC) and fronto-striatal circuits at the roots of dysfunctional self-regulation [7,8], which could underlie the lack of ability to stop overconsumption. When inhibitory control is challenged (e.g., with response inhibition tasks—see [9] for a review), differences in brain activity (functional magnetic resonance imaging, fMRI) in brain regions engaged in inhibitory control (such as PFC) seem to characterize obese and BED on one side and normal-weight individuals on the other side. Along with fMRI evidence, recent structural MRI studies supported a significant connection between eating behavior, weight condition and brain cortex morphometry in prefrontal regions [10,11,12]. Reduced gray matter volume (GMV) in the left prefrontal regions (including the dorsolateral PFC, DLPFC and Inferior Frontal Gyrus, IFG) was reported in individuals with obesity [13] and overweight women with food addiction (i.e, an addictive-like eating of highly processed food) [14]. Consistently, GMV in the middle frontal gyrus (MFG) has been found to be negatively correlated with disinhibition toward food and positively correlated with hunger scores (i.e., the intensity of hunger sensations and the extent to which such sensations induce eating) [15]. Altogether, these findings might indicate that a reduced GMV in PFC regions is linked to an enhanced tendency to lose control over food [13,14,15]. Hence, the evidence seems to speak in favor of a role of the PFC, and possibly the DLPFC, at the roots of food craving [16] and as a possible hallmark and risk factor for weight gain [11]. PFC would indeed be crucial for the ability to evaluate action consequences and, referring to eating behavior, its microstructure and functionality may have a role in the development of disordered or unhealthy eating, facilitating obesogenic habits [17,18].

A point worth noticing is that, since the majority of the evidence stems from the study of adults with obesity, overweight, and/or full-blown eating disorders, it becomes hard to disentangle possible inconsistencies in the directionality of results and to elucidate whether changes in these brain regions either act as possible neural markers and risk factors for increased propensity to disordered eating and weight gain [11,17] or rather occur as consequences of adverse metabolic factors related to overweight and obesity [19]. Hence, in the present research, we explored brain morphometry in normal-weight individuals reporting/not reporting binge-eating episodes (BE and non-BE, respectively), using a voxel based morphometry (VBM) approach. VBM is a morphometry technique that allows to explore the structure of whole brain volume voxelwise, in terms of gray/white matter and cerebrospinal fluid [20]. Here, we explored whether (i) BE and non-BE were characterized by differences in regional and global GMV. Furthermore, we determined the relation of these possible GMV differences (ii) with general impulsivity traits, as assessed by the BIS-11 [21] and (iii) self-reported binge-eating, assessed by the Binge-eating Scale (BES) [22]. 

The novelty of this study stands in the investigation of a non-clinical normal-weight population, reporting episodes of binge-eating (BE). We hypothesized that GMV differences could characterize these individuals (in respect to non-BE), even if a full-blown eating or weight disorder is diagnosed. In more detail, we expect differences between the groups to be mainly located in self-control related regions, such as the DLPFC areas, which have been repeatedly associated with impulsiveness linked to overconsumption [23]. Evidence from a non-clinical population of normal-weight binge eaters may offer fruitful insights into binge-eating mechanisms, without the possible confounding effects related to overweight or a history of eating disorder.

## 2. Materials and Methods

### 2.1. Participants

We enrolled normal-weight male and females (from 20 to 35 years old) through local advertisements at the University of Padua, and divided them in two groups taking the declared presence/absence of binge-eating episodes as a key criterion. Binge-eating status was certified by means of the Eating Attitude Test (EAT 26) [24], assessing the presence of BE episodes as well as the absence of compensatory behaviors (i.e., extreme physical activity, purging etc.). More precisely, the presence of binge-eating episodes was investigated with the following item: “I have gone on eating binges where I feel that I may not be able to stop”, scoring from 1 (never) to 6 (once a day or more) points. The following items certified the absence of purging behavior for both groups: “Ever made yourself sick (vomited) to control your weight or shape?”; “Ever used laxatives, diet pills, or diuretics to control your weight or shape?” Further, the item “Have you ever been treated for an eating disorder?” assessed the absence of a history of eating disorders. Participants declaring to have experienced at least one binge-eating episode per month within the last three months—without compensatory behaviors (i.e., excessive physical activity, purging, etc.)—constituted the binge-eaters (BE) group, while participants declaring to have never had a binge-eating episode constituted the non-BE group. To further confirm the surmised binge-eating status we used the Binge-eating Scale (BES) [22]: as an additional criteria to be included in the non-BE group, participants not reporting episodes of binge-eating were expected to obtain a score lower than 8 in the BES [25]. 

Body mass index (BMI; kg/m^2^) for participants of both groups had to range from 18.5 to 24.9 (i.e., normal-weight range according to the World Health Organization, WHO, 2013) and were right-handed according to the Edinburgh Handedness Inventory [26]. For both groups specific exclusion criteria had to be fulfilled, such as no history of psychiatric, neurological disorders, or head injuries, absence of other relevant medical issues, absence of psychoactive medication or psychotherapy. Further, all participants were checked with safety criteria for MRI examination (e.g., metal implants, pacemaker, claustrophobia, etc.). The final sample involved 21 participants for the BE group (17 females) and 21 participants for the non-BE group (16 females). The study was conducted in agreement with the guidelines provided by the Declaration of Helsinki and the ethical requirements of the University of Padua (protocol n. 2025) and informed consent was obtained from all subjects involved in the study. This study forms part of a broader line of research aiming at investigating impulsivity, brain structure and function in normal-weight BE. Participants included in the present study took part in previously published fMRI task-based and resting-state investigations [27,28].

### 2.2. Self-Report Questionnaires

Participants of both groups completed a behavioral assessment that included:

1. EAT-26 [24]: It assesses the presence of an eating disorder, by providing a measure of the symptoms and concerns that are peculiar to eating disorders. Here, we focused on the behavioral questions investigating the presence of binge-eating episodes.
2. BES [22]: It assesses the severity of binge-eating behavior relying upon both behavioral characteristics (e.g., amount of food consumed) and the emotional, cognitive responses (e.g., guilt/shame or preoccupation with food).
3. Yale Food Addiction Scale (YFAS) [29]: A 25-items self-reported assessment adopted to identify individuals prone to exhibit traits of substance addiction (in this case, consumption of high fat/high sugar foods). Items used to spot food-addiction symptoms (e.g., loss of control, tolerance, withdrawal) are built on the criteria for substance dependence as described in the DSM IV-TR [30]. Usually, three or more symptoms—plus clinically significant impairment or distress—indicates the presence of “food addiction”.
4. Barratt Impulsiveness Scale (BIS-11) [21]: It allows the investigation of three distinct forms of impulsivity: (i) attentional, (ii) motor, (iii) non-planning impulsivity.
5. Behavioral Inhibition/Behavioral Activation Scale (BIS/BAS) [31]: Investigates two complementary motivational drives controlling behavior: BIS, the aversive motivational system, sensitive to punishment/non-reward; BAS, the appetitive motivational system, sensitive to cues of reward [32].

### 2.3. Magnetic Resonance Imaging (MRI) Acquisition

MRI data were collected with a whole body 1.5 T scanner (Siemens Magneton Avanto, Erlangen, Germany) equipped with a standard Siemens eight-channels coil. High-resolution T1-weighted images were acquired (MPRAGE; 224 contiguous sagittal slices; voxel size = 0.7 × 0.7 × 0.7 mm; Field of view (FOV) 320 × 320; Matrix 320 × 295; Flip Angle (FA) 20°; Repetition Time (TR) 20 ms; Echo Time (TE) 4.89 ms; Bandwidth = 130 Hz).

### 2.4. MRI Analysis

MRI data preprocessing and analysis were performed using a VBM approach [20]. Preprocessing was conducted using the Computational Anatomy Toolbox (CAT12–Department of Neurology, Jena University Hospital, Germany-http://dbm.neuro.uni-jena.de/cat/, accessed on 31 August 2021), an extension of SPM12 (Statistical Parametric Mapping-The Wellcome Centre for Human Neuroimaging, UCL Queen Square Institute of Neurology, London, UK-https://www.fil.ion.ucl.ac.uk/spm/software/spm12/, accessed on 31 August 2021) implemented in MATLAB 2011a environment (update 7.12.0—powered by Mathworks, Natick, MA, USA). Default settings provided by CAT12 were adopted. Preprocessing of MR images included (i) normalization according to the Montreal Neurological Institute (MNI) template; and (ii) segmentation into gray matter (GM), white matter (WM), and cerebrospinal fluid (CSF). After these steps, (iii) a quality check was performed and, the images were smoothed with a Gaussian kernel of 8 × 8 × 8 mm Full Width at Half Maximum (FWHM). The total intracranial volume (TIV) and percentage of global tissue volumes (GM, WM and CSF) of each subject were computed with CAT 12 and compared between the two groups (BE and non-BE) with the software Statistical Package for Social Sciences, version 23 (SPSS23, powered by the International Business Machine Corporation–IBM–Armonk New York, USA-https://www.ibm.com/support/pages/downloading-ibm-spss-statistics-23/, accessed on 31 August 2021).

The assessment of between-group differences focused only on GMV: we used GM images of each participant and performed voxel-wise two-samples *t*-tests within a General Linear Model (GLM) in SPM 12, using a Whole Brain approach. In order to control for the possible influence of difference in TIV and age, these variables were included as covariates of no interest in the statistical model. Besides, given that sex/gender differences in both brain activity and structure has been well documented in healthy adults and individuals with obesity (for review see [33]), we also included gender as a covariate of no interest in the statistical model. Resulting statistical maps were first thresholded at a whole-brain voxel-wise level (<0.001 uncorrected); the results were then thresholded on a cluster level (Family Wise Error, FWE < 0.05). Only the surviving clusters are reported.

### 2.5. Brain-Behavior Correlations

In order to investigate the possible relations between brain morphometry, trait impulsivity, and binge-eating behavior, the mean GMV values were extracted from the cluster showing differential GMV between the groups (i.e., results from the t-test comparisons) with the SPM toolbox “MarsBaR” [34], and correlated with BIS-11 and BES scores. Based on the distribution of the data and the variables considered, we conducted non-parametric correlation analyses (Spearman’s rho) in SPSS 23. 

## 3. Results

### 3.1. Descriptive Statistics

Table 1 shows descriptive characteristics of the participants and the self-report questionnaires’ scores. The two groups did not differ for age, sex (~30% males), and BMI, but they did in most of the questionnaires’ total and subscales’ scores (see Table 1). The BE group was characterized by higher scores in the BES, YFAS, and BIS 11, whereas non-BE had higher scores for the BIS and the BAS drive subscales of the BIS/BAS questionnaire. Hence, BE, compared to non-BE, showed enhanced general trait and food-related impulsivity (as assessed by BES, YFAS, and BIS-11), but did not show a greater sensibility to general rewards (as indicated by the BAS subscale: “reward responsiveness”).

### 3.2. Voxel-Based Morphometry (VBM)

#### 3.2.1. Global Volumes: Between-Groups Comparison

Percentage of global volumes of GM, WM, CSF, and TIV did not differ between the groups (Table 2).

#### 3.2.2. Whole Brain Analysis: Between-Group Comparison

**Figure 1:**
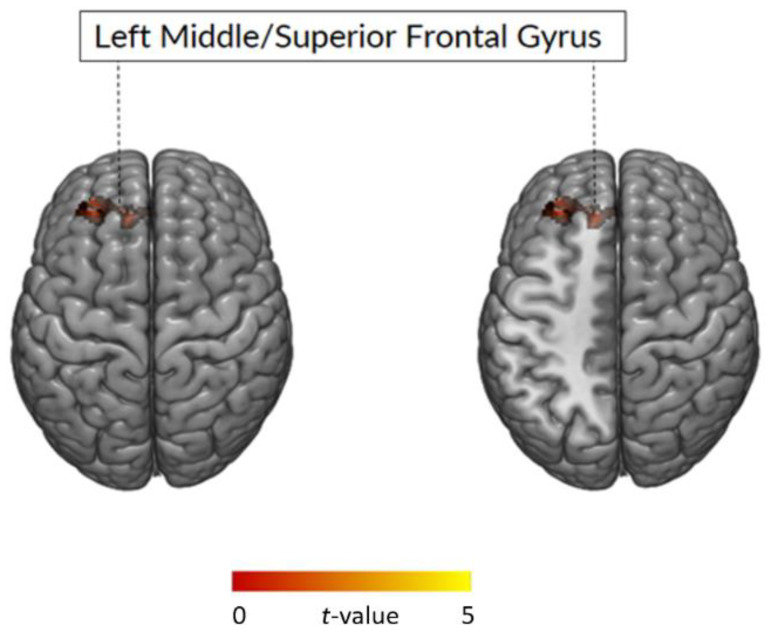
Whole Brain Analysis: BE > non-BE comparison. Results for the BE > non-BE comparison (MNI: −26; 44; 35, Table 3). Statistical parametric maps are overlaid onto a Montreal Neurological Institute (MNI) template provided by the software MRIcroGL. The color-bar is representative of the *t*-scores reported in Table 3. Images are shown in neurological convention (i.e., left side of the image corresponds to the left side of the brain). Notes: BE = binge eaters; non-BE: non binge eaters.

Increased GMV in the left middle and superior frontal gyrus (MFG and SFG, respectively) were observed in the BE group when compared to the non-BE. The opposite comparison (non-BE > BE) did not yield any significant result (Figure 1; Table 3). Figure 1 illustrates the regions where significant differences were observed between BE and non-BE.

#### 3.2.3. Correlation between GMV and Impulsivity Traits

The correlations between GMV in the left MFG and scores of the BIS-11 questionnaires (attentional, motor, non-planning and total scores) did not yield any significant results in both groups (Table A1 and Table A2).

#### 3.2.4. Correlation between GMV and Binge-Eating Behavior

**Figure A1:**
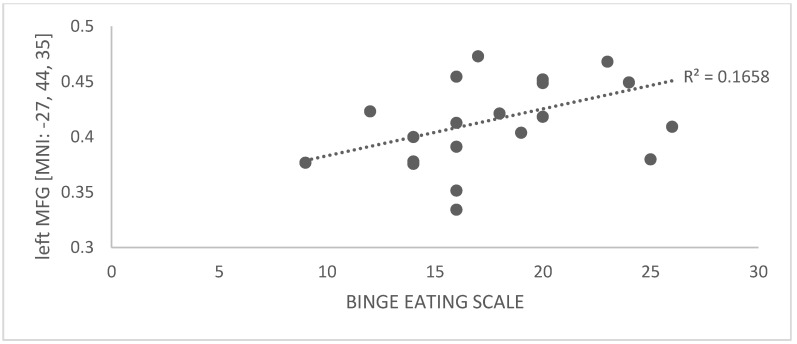
Correlation between BES scores and GMV in the left MFG, in the BE group. Notes: BES: binge-eating scale; GMV: gray matter volume; MFG: middle frontal gyrus; MNI: Montreal Neurological Institute.

The correlational analysis revealed a significant positive correlation between the extracted GMV within the cluster in the left MFG and the scores of the BES (Table 4; Figure A1) in the BE group but not in the non-BE group (Table A1).

## 4. Discussion

The present research aimed at exploring the possibility that normal-weight BE—compared to non-BE—would be characterized by differences in GMV, especially in PFC, assumed to have a role in behavior regulation [4].

BE, compared to non-BE, showed higher BIS-11 scores, indicating high general trait impulsivity, and higher scores in the BES and YFAS, suggesting the presence of loss of control and addictive-like tendencies toward food. In addition, BE showed lower scores for the BIS subscale of the BIS/BAS questionnaire [27]. This subscale explores the regulation of aversive behavior, and higher scores are usually associated to a tendency to avoid aversive stimuli [31]. Evidence indicates BIS score increases as a function of restraint [35,36]; as a consequence, lower scores might indicate a weakened tendency to avoid or inhibit behavior, with resulting greater propensity to respond. Altogether, these findings support the premise of impulsive traits having a role at the roots of loss of control behavior and compulsive consumption [6], even no eating or weight disorders are diagnosed.

With regard to brain morphometry, results indicated that BE and non-BE did not differ in terms of global volumes but differed in the regional GMV. In line with our hypotheses, BE—compared to non-BE—showed higher GMV in the left MFG and partially, the SFG. The MFG—together with the SFG—belongs to the DLPFC, a brain area involved in executive control processes [37]. DLPFC is assumed to have a role in behavioral control, and therefore in mediating impulsive behaviors that are described as peculiarity of the binge-eating conduct (e.g., impulsivity, poor self-regulation/decision-making) and that are usually tied to overeating and weight gain [38,39]. Support for the involvement of the DLPFC in overeating stems from several neuroimaging studies showing changes within this region in obesity and binge-eating syndromes [9,23,40]; DLPFC involvement has been reported in response to food pictures in individuals with high food addiction symptoms [41] and during the inhibition of an already initiated response in binge-eating [17,27]. In particular, a higher activity of the left MFG during a food-specific response inhibition task with food cues has been described in a study considering the same sample of normal-weight BE [27].

The involvement of the left DLPFC has been previously described in overeating as a key region for inhibitory control processes [42]; for instance, low levels of activation in the left DLPFC have been linked to impaired goal-oriented behavior and uncontrolled eating in obesity [23]. Whereas, the MFG regional volumes have been associated to disinhibition toward food in normal and over-weight adults [14] and with increased BMI after one year in young females, suggesting a role of this region in weight gain [43]. The reason for our results showing greater GMV in this region, however, deserves further exploration. Indeed, structural MRI studies have also described results in the opposite direction (i.e., decreased GMV in the DLPFC in overweight and overeating conditions) [40]. From a functional point of view, since the stronger the request for inhibitory control, the greater the activation in prefrontal regions [44], a stronger recruitment of prefrontal circuits may underlie an additional engagement of resources in order to exert self-regulation and suppress urges [17]. On the structural side, relying on the evidence of positive correlations between GMV and brain activity [45], we might speculate that greater GMV of the left MFG in BE could support the idea of its involvement in self-regulatory mechanisms toward food. Interestingly, greater GMV of left MFG has been also described in binge drinking [46,47]: even if binge drinking and binge-eating are two phenotypically different conditions, they are thought to share—at least partially—common neural and behavioral substrates [48]. Hence, higher GMV within the MFG might underly the tendency to act impulsively and the need to exert additional self-control, regardless of the reward involved (i.e., food or alcohol).

Contrary to our expectations, we did not find any significant correlation between the GMV in the left MFG and the BIS-11 scores, while a positive correlation with BES scores was observed. This result suggests that the binge-eating status may—at least partially—account for the structural differences between BE and non-BE in the left MFG. Although the directionality of these results needs to be interpreted with caution and deserves further investigation, our result points toward the intriguing possibility of a close connection between the tendency to overeat and structural GMV changes in left DLPFC in BE, even if in a condition of normal weight. On the other hand, the lack of significant correlations with general impulsivity might lie on the self-report measure we adopted: even though the BIS-11 is a reliable tool to assess trait impulsivity, impulsivity is known to be a multidimensional construct [49]. Thus, the use of different self-report and behavioral measures to assess other facets of impulsivity (e.g., positive/negative urgency, sensation seeking, etc.,) might help elucidate this finding.

## 5. Conclusions

Overall, BE—compared to non-BE—showed higher GMV in the left MFG, positively correlated with binge-eating behavior. These results, together with the involvement of prefrontal regions in the characterization of overeating conditions [40], may add support to the idea of eating behavior and body weight as partly subserved by higher-level processes involved in cognition, decision-making, and motivation [12]. Most importantly, our results in a non-clinical population of normal-weight BE deepen the understanding of overeating correlates, excluding the possible weight-related confounding effect on results’ interpretation. Nonetheless, some limitations need to be acknowledged. Since the DLPFC is involved in a number of processes, a part from inhibitory control (e.g., working memory, cognitive flexibility, planning, etc.), a more complete investigation of the different facets of impulsivity could help outline the nature of the differences in brain morphometry and the underlying mechanisms related to binge-eating. Within this context, accounting for lifetime binge-eating episodes and co-occurring comorbities (e.g., other-than-eating addictions, mood and anxiety disorders) may unravel additional insights. Furthermore, given the cross-sectional nature of our study, we are not able to infer any cause-effect relationships between brain structure and eating behavior. Thus, future longitudinal studies are needed to elucidate whether GMV of the DLPFC may represent a stable feature of binge-eating, and act as possible risk factors for the development of eating and weight disorders, as well as a ground for targeted prevention and treatment interventions.

## Figures and Tables

**Table 1:** Descriptive characteristics: between-group comparisons for age, body mass index and self-report measures.

Characteristics	BE (*n* = 21) M ± SD	NON-BE (*n* = 21) M ± SD	Two-Samples *t*-Test	
AGE	23.9 + 3.19	25.23 ± 3.08	2.05	0.191
BMI (kg/m^2^)	22.3 ± 2.1	21.29 ± 2.02	1.73	0.074
BES	17.7 ± 3.8	3.8 ± 2.6	17.1	<0.001 *
YFAS	3.05 ± 1.43	0.29 ± 0.56	8.23	<0.001 *
BIS-11				
Subscale ATTENTION	17.05 ± 3.7	15 ± 3.3	1.8	0.075
Subscale MOTOR	20.73 ± 4.2	17.75 ± 3.3	2.5	0.015 *
Subscale NON-PLANNING	26.32 ± 5.1	22.25 ± 4.1	2.8	0.007 *
TOTAL SCORE	63.4 ± 8.8	56 ± 7.5	2.7	0.011 *
BIS/BAS				
BAS reward responsiveness	7.3 ± 1.8	7.6 ± 2.1	0.43	0.075
BAS Drive	7.8 ± 1.7	9.3 ± 1.9	2.51	0.017 *
BAS Fun seeking	8.7 ± 2.1	9.4 ± 2.4	1.03	0.13
BIS	13.3 ± 2.3	16.2 ± 3.6	2.88	0.007 *

Notes: M = mean; SD = standard deviation; *t* score and *p*-value. BMI: body mass index; BES: Binge-eating Scale; YFAS: Yale Food Addiction Scale; BIS-11: Barratt Impulsiveness Scale; BAS: behavioral activation system; BIS: behavioral inhibition system; Non-BE: non-binge eaters; BE: binge eaters. * Correlation is significant at the level of 0.05.

**Table 2:** Global Volumes: between-groups comparison for global volumes values (gray matter, white matter, cerebrospinal fluid) and total intracranial volume (mL).

	Non-BE	BE	Two Sample *t*-Test	
	M ± SD	M ± SD	t	*p*
(%) GM	19.4 ± 7	19.6 ± 3.3	0.11	0.915
(%) WM	44.6 ± 1.8	44.1 ±3.4	0.62	0.537
(%) CFS	37 ± 1.3	36.3 ± 1.6	1.59	0.12
TIV (mL)	1422.4 ± 142.4	1483.2 ± 134.4	1.42	0.162

Notes: M: mean; SD: standard deviation; t: *t*-score; BE: binge eaters; non-BE: non binge eaters; GM: gray matter; WM: white matter; CSF: cerebrospinal fluid; TIV: total intracranial volume.

**Table 3:** Whole brain analysis: two sample *t*-test (covariates: age, gender and total intracranial volume).

Whole Brain Analysis						
k	*p* (FWE)	t	z-Score	MNI	Side	Region
BE > non-BE						
604	0.002	4.92	4.29	−27, 44, 35	L	Middle Frontal Gyrus
		4.20	3.77	−15, 41, 29	L	Superior Frontal Gyrus
		3.89	3.54	−35, 41, 41	L	Middle Frontal Gyrus
Non-BE > BE						
*ns*						

Notes: K = number of voxels; t and z scores; stereotaxic coordinates according to the MNI space; brain side and region. Statistic threshold: Results were considered significant at *p* < 0.001 uncorrected that additionally met a FWE correction at cluster level (*p* < 0.05). BE: Binge Eaters; non-BE: non-binge eaters; FWE: family wise error; L: left; R: right; MNI: Montreal Neurological Institute.

**Table 4:** BE group: correlation between BES scores and GMV in the left MFG.

	Extracted GMV in Left MFG	
	Spearman’s Rho	*p*-Value
BES	0.463	0.035 *

Notes: BES: Binge-eating Scale; GMV: gray matter volume; MFG: middle frontal gyrus; MNI: Montreal Neurological Institute. * Correlation is significant at the level of 0.05 (2-tailed).

## Data Availability

The data presented in this study are available on request from the corresponding author. The data are not publicly available due to data ownership regulations and privacy regulations contained in the informed consent signed by participants involved in the study.

## Appendix A

**Table A1:** Non-BE group: Correlation between BIS-11 and BES scores and GMV in the left MFG.

Extracted GMV in Left MFG [MNI: −27, 44, 35]		
	Spearman’s Rho	*p*-Value
BIS-11 Total score	−0.048	0.835
Subscale ATTENTION	0.107	0.644
Subscale MOTOR	−0.264	0.247
Subscale NON-PLANNING	0.047	0.839
BES	0.257	0.261

Notes: BIS-11: Barratt Impulsiveness Scale; BES: Binge Eating Scale; GMV: Gray Matter Volume; MFG: Middle Frontal Gyrus; MNI: Montreal Neurological Institute.

**Table A2:** BE group: Correlations between BIS-11 and GMV in the left MFG.

Extracted GMV in Left MFG [MNI: −27, 44, 35]		
	Spearman’s Rho	*p*-Value
BIS-11 Total score	0.150	0.518
Subscale ATTENTION	0.289	0.204
Subscale MOTOR	0.084	0.719
Subscale NON-PLANNING	0.037	0.872

Notes: BIS-11: Barratt Impulsiveness Scale; GMV: gray matter volume; MFG: middle frontal gyrus; MNI: Montreal Neurological Institute.
